# Co–Sn–Cu oxides/graphene nanocomposites as green catalysts for preparing 1,8‐dioxo‐octahydroxanthenes and apoptosis‐inducing agents in MCF‐7 human breast cancer cells

**DOI:** 10.1049/nbt2.12006

**Published:** 2021-02-22

**Authors:** Kaveh Parvanak Boroujeni, Zeinab Tohidiyan, Zahra Lorigooini, Zahra Hamidifar, Mohammad Mehdi Eskandari

**Affiliations:** ^1^ Department of Chemistry Shahrekord University Shahrekord Iran; ^2^ Department of Chemistry Shahrekord Branch Islamic Azad University Shahrekord Iran; ^3^ Medical Plants Research Center Basic Health Sciences Institute Shahrekord University of Medical Sciences Shahrekord Iran; ^4^ Nanotechnology Research Center Research Institute of Petroleum Industry Tehran Iran

## Abstract

In this work, Co–Sn–Cu oxides/graphene nanocomposite, 30–40 ± 0.5 nm in size, was synthesized by solid‐state microwave irradiation. This method presents several advantages such as operational simplicity, fast, low cost, safe and energy efficient, and suitability for production of high purity of nanoparticles. Other advantages of this method are there is no need for the use of solvent, fuel, and surfactant. Co–Sn–Cu oxides/graphene nanocomposites have been characterized by Fourier transform infrared spectroscopy, X‐ray diffraction, field emission scanning electron microscopy, transmission electron microscopy, vibrating sample magnetometer, energy‐dispersive X–ray spectroscopy, and UV–Vis spectroscopy. The synthesized nanocomposites were used as novel highly efficient catalysts for the synthesis of 1,8‐dioxo‐octahydroxanthenes at room temperature. The catalysts are recoverable and can be reused for six runs without loss of their activity. Also, the obtained nanocomposites exhibited significant anticancer activity against breast cancer cells and they could induce apoptosis in cancer cells.

## 1 INTRODUCTION

Graphene and graphene oxide (GO) have been widely used to fabricate various kinds of composite materials and also for catalysis synthesis due to their high surface area, high chemically stability, strong conductivity for electron transfer, and low cost [1, 2]. Among these, graphene supported metal nanocomposites has received more attention [3]. For example, bimetallic Sn‐W oxides/graphene and tin–zirconia/grapheme nanocomposites have been reported as heterogeneous catalysts for selective oxidation of alcohols [4] and synthesis of dimethyl carbonate [5], respectively. However, despite many examples of immobilized metal nanocomposites, little is established on their usage as a catalyst in organic transformations.

**SCHEME 1:**
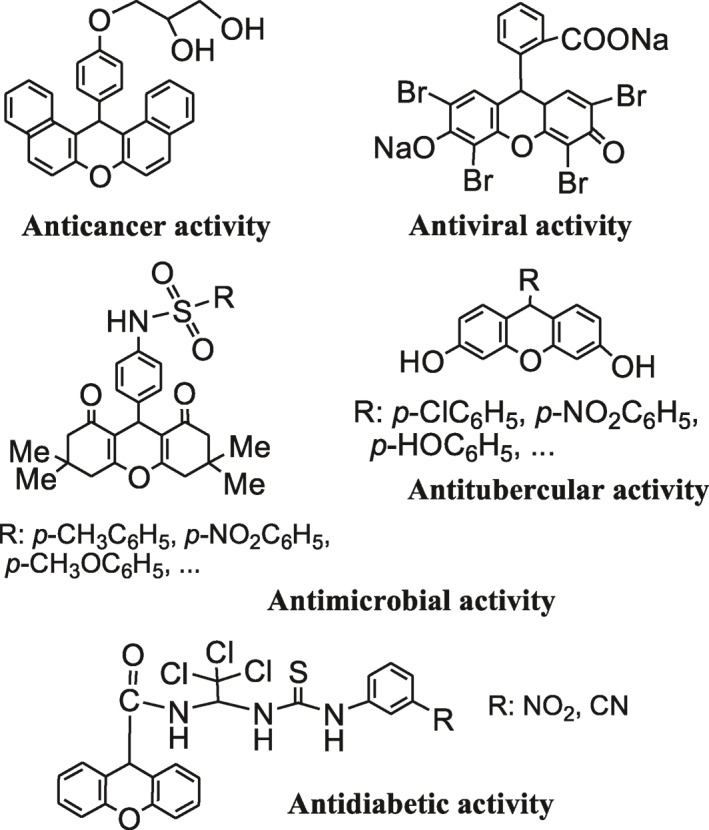
Biological applications of a series of xanthene derivatives

Natural products with a xanthene moiety have revealed biological activities such as anti‐inflammatory [6], anti‐bacterial [7], and anti‐cancer activities [8] (Scheme 1). 1,8‐Dioxo‐octahydroxanthenes are one of the most important derivatives of xanthenes which were usually synthesized via one‐pot Knoevenagel condensation, Michael addition, and cyclodehydration of 5,5‐dimethyl‐1,3‐cyclohexanedione (dimedone) with various aldehydes. Numerous Lewis and Brönsted acid catalysts have been used to promote this reaction [9, 10, 11, 12, 13, 14, 15, 16, 17, 18, 19, 20]. Although these catalysts are suitable for certain synthetic conditions, their main disadvantages are long reaction times, tedious work‐up, the formation of uncyclized adduct 2,2’‐aryl‐methylene bis(3‐hydroxy‐2‐cyclohexene‐1‐one), and the use of unrecyclable, hazardous, or difficult to handle catalysts. The need to high temperatures [9, 10, 11, 12, 13, 14, 15, 16] or an additional energy (ultrasound or microwave) [17, 18] is another drawback of many of these methods. These reactions are rarely carried out at low temperature [19, 20]. Hence, the development of new catalysts with more efficiency is still in demand.

Transition metals have been received much attention from the biological point of view because they are the key linkers of complex proteins or enzymes [21, 22]. The mechanism of action of the bivalent cationic metalloestrogens in breast cancer has been reviewed recently [23]. Metal‐based compounds including copper and tin have been surveyed with promising chemotherapeutic potential for the treatment of a range of cancers [24, 25, 26, 27]. Because of the great potential of copper and tin complexes as anticancer agents, complexes made up of both metals have attracted much attention due to a plausible dual mode of action at the molecular level [28]. For instance, the chiral l‐tryptophan‐derived [bis(1,2‐diaminobenzene) copper(II)] chloride complex, CuSn_2_(Trp), showed antitumor activity [29] as well as induced cytotoxicity in PA‐1, PC‐3, HeLa, U2OS, HepG2, and MCF‐7 cancer cell lines [30]. In addition, cobalt‐based ligand complexes have a wide spectrum of biological activities as antibacterial and antiviral properties [31]. Cobalt‐doped magnetite, iron–cobalt/ferrite core–shell structures, and cubic iron–cobalt nanoparticles have been used for hyperthermia application in cancer therapy [32, 33, 34]. Also, the efficiency of graphitic carbon–cobalt nanoparticles as localized radio frequency absorbers for cancer treatment has been investigated [35].

Breast cancer is one of the main health concerns facing our society and is the second leading cause of morbidity and mortality among women worldwide [36]. For clinical treatment of breast cancer, in many occasions chemotherapy is one important option. Nanotechnology is on the route to providing novel developments in medicine science, such as designing biocompatible inorganic nanomaterials with applications of determination of drug [37] and anti‐oxidant [38], anti‐bacterial [39], and anti‐biofilm properties [40]. In addition, nanotechnology has attracted attention in tumor imaging and cancer therapy and in this regard nanoparticles with many unique properties, such as a high surface area‐to‐volume ratio and magnetic and optical properties, play important roles [41]. In this regard, the graphene‐based nanomaterials have attracted considerable interest in upcoming nanotechnology‐based medicine applications [42]. For instance, GO as a carrier for adriamycin in cancer drug resistance [43] and the effect of curcumin‐reduced GO on breast cancer cells [44] have been investigated. GO–silver nanocomposite [45] and gallic acid/CdS on the GO nanosheets [46] were used for targeted therapy of human ovarian cancer stem cells and treatment of kidney tumor cells, respectively. Tamoxifen citrate on reduced GO nanosheets was used for breast cancer therapy [47]. Also, Zhou et al. applied platinated GO nanostructure for efficient gene‐chemo combination cancer therapy [48]. The hydrophilic graphene‐based yolk‐shell magnetic nanoparticles functionalized with copolymer pluronic F‐127 (GYSMNP@PF127) has been reported as an efficient multifunctional biomedical system for mild hyperthermia and stimuli–responsive drug delivery [49].

Various synthetic techniques have been reported for preparing metal nanocomposites, including sonochemical [50], thermal decomposition [51], hydrothermal [52], and sol‐gel methods [53]. In this regard, the important issue for choosing synthetic method is reducing the cost of physical and chemical synthesis route; meanwhile, it can produce nanocomposites with high purity in short time. Solid‐state microwave‐assisted method with controlling time and reaction temperature quite provides these main purposes and is able to generate nanocomposites in narrow particle size distribution [54]. Along this line and in the continuation of our recent works on synthesis of NiO nanoparticles using solid‐state microwave‐assisted method [55] and investigation of biological activity [39, 56, 57] and drug determination [37] of the synthetic compounds, the synthesis of Co–Sn–Cu oxides/graphene nanocomposites by solid‐state microwave irradiation and their use as novel catalysts for preparing 1,8‐dioxo‐octahydrozantenes are reported. Also, the anti‐proliferative activity and apoptosis induction of nanocomposites on a breast cancer cell line is investigated.

## 2 EXPERIMENTAL SECTION

### 2.1 Materials and methods

All chemicals were either prepared in our laboratory or were purchased from Merck and Fluka. Reaction monitoring and purity determination of the products were accomplished by gas liquid chromatography or thin‐layer chromatography (TLC) on silica‐gel polygram SILG/UV254 plates. Gas chromatography was carried out on Shimadzu GC 14–A. ^1^H NMR spectra were recorded on 400 MHz spectrometer in CDCl_3_. Melting points were determined on a Fisher–Jones melting‐point apparatus. To determine the crystalline phase, powder X‐ray diffraction (XRD) analysis was carried on an X‐ray diffractometer with Ni‐filtered Cu Kα irradiation (λ = 1.54 A°), for an angle range of 2θ = 10–80°. Infrared spectrum was carried on a Fourier transform infrared (FT–IR) 160 spectrophotometer (Shimadzu system) by KBr tablets. UV–Vis spectroscopy measurement at room temperature (298 K) was obtained by a double‐beam Shimadzu 1650 PC. The powder samples for UV–Vis analysis were dispersed in EtOH for 25 min to make a homogeneous suspension. Field emission scanning electron microscopic (FESEM) images were taken on a Hitachi s4160/Japan equipped with a link energy dispersive X‐ray (EDX) analyzer with gold coating. Transmission electron microscopy (TEM, Philips CM10) was applied at accelerating voltage of 300 kV. The sample was sonicated in EtOH, and a drop of the suspension was dried on a carbon‐coated microgrid for the TEM analysis. The magnetic property of the sample was measured at room temperature (298 K) using a vibrating sample magnetometer (VSM, MDKFD, Meghnatis Kavir Kashan Co.). Ultrasonic generator was carried out on ultrasonic probe (Top‐Sonics UPH‐400, ultrasonic technology development Co.). A microwave oven (LG, 2.45 GHz, 900 W) was used for the microwave irradiation. For anticancer study, data were analyzed by SPSS using the Kruskal–Wallis test and are presented as the means ± standard deviation from at least three independent experiments. The apoptosis was examined by flow cytometry (Partec System, Germany). IC50 value was calculated using probit analysis.

### 2.2 Synthesis of Schiff base ligand (H2L)

4,4’‐Dibromo‐2,2’‐[cyclohexane‐1,2‐diylbis(nitrilomethanylylidene)]diphenol (H2L) was synthesized according to our previous study [56]. 1,2‐Diaminocyclohexane (0.1 g, 1 mmol) was added to 5‐bromosalicylaldehyde (0.4 g, 2 mmol) in 20 mL MeOH. The mixture was refluxed for 1 h. The yellow powder was filtered off, washed with cold methanol, and dried under vacuum over anhydrous CaCl_2_.

### 2.3 Synthesis of precursor powders

Three solutions of H2L ligand (0.3 mmol, 0.15 g) in methanol (10 mL) were exposed to ultrasonic irradiation, and 10 mL of a methanolic solution of Cu(CH_3_COO)_2_.2H_2_O (0.14 g. 0.3 mmol) was added to each solution in a dropwise manner [57]. Then, 0.1 mmol of Co(NO_3_)_2_.5H_2_O with different amounts of SnCl_2_.2H_2_O (0.005, 0.01, or 0.02 mmol) dissolved into 20 mL of methanol was added to each above mixture under ultrasonic irradiation (300 W) for 20 min at room temperature to form a homogeneous light brown solution. When the pH of each mixture was adjusted to 11 by NaOH aqueous solution (1 M), a precipitate was formed which was then filtered off, washed with cold methanol, and dried under vacuum over anhydrous CaCl_2_. Three obtained powders were used as precursors for the preparation of corresponding Co–Sn–Cu oxides/graphene nanocomposites.

### 2.4 Synthesis of Co–Sn–Cu oxides nanocomposites

To prepare Co–Sn–Cu oxides/graphene nanocomposites, in separate tests, 2 g of the above precursor samples was added to a porcelain crucible and each one was placed in another bigger porcelain crucible that is filled with CuO powder (microwave absorber). The assembly was placed in a microwave oven and exposed to microwave irradiation at 900 W (100%) in air. Along irradiation, temperature of CuO powder increased from room temperature to 450ºC. The temperature was determined by a chromel–alumel thermocouple in the mixture vessel. The generated heat was transferred to precursor sample and it was decomposed. After 10 min, decomposition of each powder was completed. The obtained products were cooled to room temperature, washed with ethanol, and dried under vacuum over anhydrous CaCl_2_ to produce three Co–Sn–Cu oxide nanocomposites.

### 2.5 Synthesis of GO

A double‐jacket reactor was charged with 85% phosphoric acid (10 mL), and the circulator temperature was adjusted at 5°C. Then, concentrated sulfuric acid (90 mL) was added to the mixture and stirred. A well‐ground mixture of graphite (1 g, particle size <50 µm) and potassium permanganate (5 g) was gradually added into the above solution over a period of 2 h, and the mixture was stirred at 5°C for 3 h. Afterwards, the mixture was stirred with stepwise temperature rise to 25°C for 3 h and then to 55°C in 2 h. After that, the mixture was stirred at 55°C for 12 h, cooled to 5°C, and finally poured into crushed ice and stirred. To the resulting mixture, hydrogen peroxide (30%) was added slowly until an ochre color was achieved. The precipitate formed was collected by filtration, washed twice with 50 mL of deionized water and then with concentrated hydrochloric acid (2 × 10 mL). The washing was repeated with deionized water until sulfate and manganese ions were no longer present in the eluate (checked by barium and sodium bismuthate tests). Finally, the GO product was washed with ethanol and acetone and dried under vacuum at 70°C overnight [58].

### 2.6 Synthesis of reduced GO from GO

Reduced graphene oxide (rGO) was synthesized through the previously reported method [59]. To a suspension of GO (500 mg) in water (20 mL) was added 50 mL of hydrazine monohydrate. The mixture was refluxed for 24 h at 100ºC. The obtained powder was washed with water and ethanol and dried under vacuum over anhydrous CaCl_2_.

### 2.7 Synthesis of Co–Sn–Cu oxides/graphene nanocomposites (1, 2, and 3)

In separated tests, to 20 mL of ethanolic suspension of the three above prepared Co–Sn–Cu oxides nanocomposites (0.1 g), 10 mL of ethanolic solution of rGO (0.1 mg) was added dropwise and stirred at 90ºC for 30 min and it was exposed to ultrasonic irradiation (250 W) for 10 min. The formed powders were separated by centrifugation and dried at 120ºC for 12 h. The obtained products were named nanocomposites 1–3 based on tin concentration of 0.005, 0.01, and 0.02 mmol, respectively.

### 2.8 Typical procedure for preparing 1,8‐dioxo‐octahydroxanthenes

A mixture of 3‐nitrobenzaldehyde (1 mmol), dimedone (2 mmol), nanocomposite 3 (0.01 g), and ethanol/water (3 mL, 1:1) was prepared and stirred at room temperature. After completion of the reaction (monitored by TLC), the catalyst was removed by an external magnet and washed with 5 mL of ethanol and the filtrate was concentrated on a rotary evaporator under reduced pressure to yield the corresponding product. Whenever required, the products were purified by recrystallization from ethanol.

### 2.9 Cell culture and treatment

Normal and human breast cancer cells (MCF‐7) were obtained from National Cell Bank of Iran (NCBI), Pasteur Institute of Iran (Tehran, Iran). The MCF‐7 cells were cultured in standard condition (95% humidity, 5% CO_2_, 37ºC) in RPMI 1640 supplemented with 10% heat‐inactivated fetal bovine serum. A 200 mM solution of each nanocomposite (1–3) in 100% dimethyl sulfoxide (DMSO) and different concentrations was prepared with the RPMI 1640; the final concentration of DMSO was less than 0.02% in both control and treated cells.

### 2.10 Cell viability assay

MCF‐7 were seeded in a concentration of 8 × 10^3^ cells per well in 96–well plates for 24 h before treatment and exposed to different concentrations of nanocomposites (0–500 μg/mL) for 24 and 48 h. Cell viability was then examined by the colorimetric assay using the 3‐(4,5‐dimethylthiazol‐2‐yl)‐2,5‐diphenyltetrazolium bromide (MTT) assay, according to the manufacturer's instruction (Sigma Aldrich, USA). Briefly, after different incubation times, cells were washed twice with phosphate‐buffered saline (PBS) and MTT solution prepared in PBS (5 mg/mL) was added to each well to the final concentration of 0.5 mg/mL in no‐phenol red RPMI and was incubated for 3 h in a dark place. After washing with PBS, 100 μL of DMSO was added to each well, and absorbance was measured at 570 nm by an Elisa reader (stat fax‐2100 awareness). Viably of each group was measured according to the absorbance compared with control.

### 2.11 Apoptosis assay

MCF‐7 cells were seeded in 6–well plates at a density of 1 × 10^6^ cells per well, grown for 24 h before treatment with nanocomposites at IC50 value for 48 h. The apoptosis was examined by flow cytometry using the Annexin V‐FITC/propidium iodide (PI) apoptosis kit (BD Biosciences), according to the manufacturer's instruction. Briefly, MCF‐7 cells were washed with cold PBS and were then resuspended in 1 mL of 1x binding buffer (provided with kit) at density of 10 × 10^6^ cells/mL. 100 µL of cell suspension was incubated with 5 µL of Annexin V‐FITC and PI for 20 min in the dark at room temperature, and then examined by flow cytometry. The percentage of cells in different stages (apoptosis and necrosis) was examined by FlowJo 7.6 software. At least 12,000 events were recorded and represented as dot plots.

## 3 RESULTS AND DISCUSSION

### 3.1 Synthesis and structural characterization of nanocomposites

**SCHEME 2:**
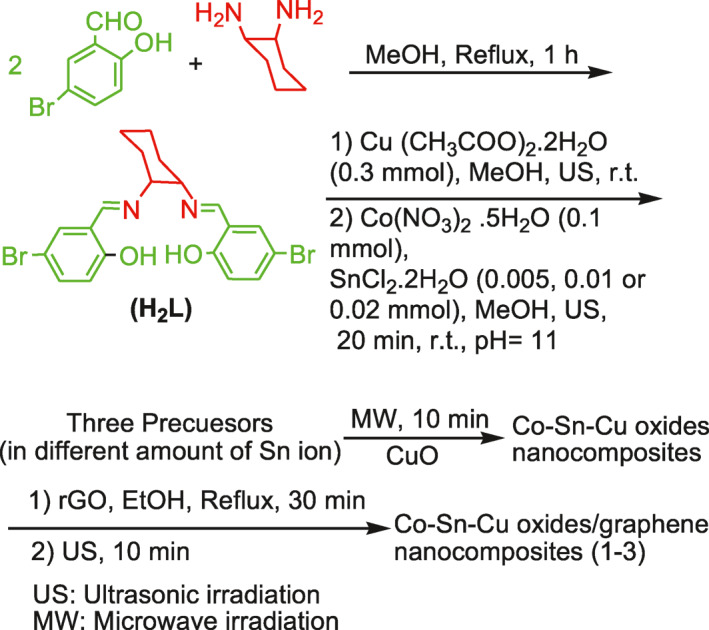
The synthetic routes for Co–Sn–Cu oxides/graphene nanocomposites

By solid‐state microwave irradiation, three nanocomposites (1, 2, and 3) were prepared in a short time (Scheme 2). For this purpose, first a Schiff base ligand (H_2_L) was prepared using 1,2‐diaminocyclohexane and 5‐bromosalicylaldehyde which was then reacted with Cu(CH_3_COO)_2_.2H_2_O, Co(NO_3_)_2_.5H_2_O, and SnCl_2_.2H_2_O at different concentrations to produce three precursors. Afterwards, each precursor was exposed to microwave irradiation to yield their corresponding nanocomposites. However, we observed that the precursors remained unchanged for 30 min, indicating that they do not absorb microwaves energy. Therefore, reaction requires a secondary irradiation absorber. For this purpose, CuO powder was used. Then, each precursor was completely decomposed through the absorption of heat from the hot CuO and the corresponding nanosized Co–Sn–Cu oxides/graphene nanocomposites were formed. Decomposition of each precursor was companied by the release of different gases such as N_2_, NO, N_2_O, and H_2_O vapors. After the synthesis of nanocomposites, the Co–Sn–Cu oxides/graphene nanocomposite 3 (with 0.02 mmol of Sn ion, abbreviated hereinafter to nanocomposite 3) was considered as representative sample for characterization by different analyses.

**FIGURE 1:**
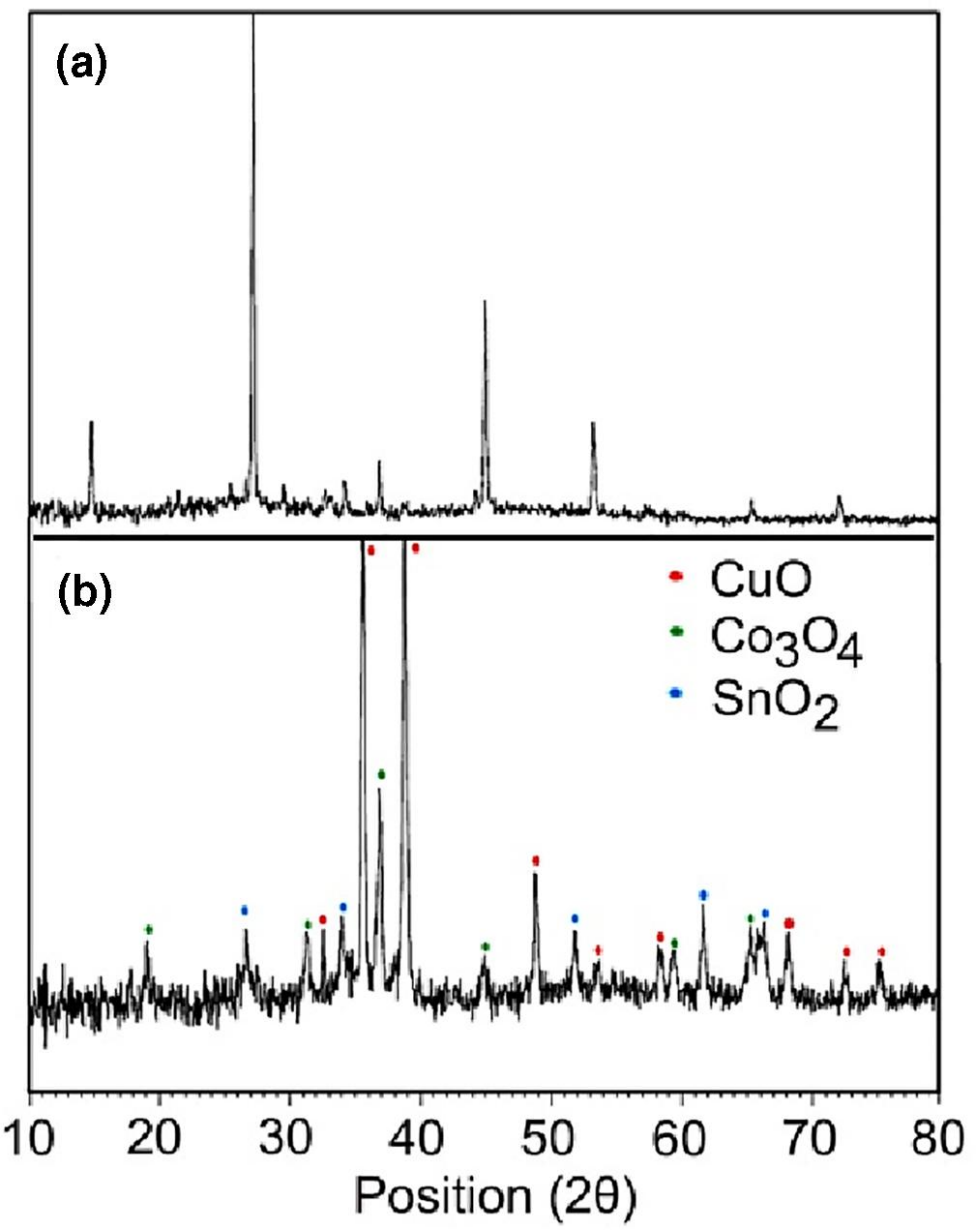
XRD pattern of nanocomposite 3 (b) and its corresponding precursor (a)

Figure 1(a) shows XRD pattern of the corresponding precursor for nanocomposite 3. This pattern manifested that the particles were in nanometer scale. The main diffraction peaks were observed at 2θ = 27.1705° and 45.0807°. The average particle size was calculated to be about 42.72 nm, by applying a full width at half maximum and the value of 2θ of the characteristic peak of the XRD pattern using the Debye–Scherrer equation [60]. Figure 1(b) displays the XRD pattern of nanocomposite 3 at the power level of 900 W for 10 min in a microwave cavity. All the diffraction peaks of the XRD pattern could be indexed to the monoclinic CuO phase (space group: C2/c; No. 15, JCPDS Card No. 80‐1268) at 2θ = 35.5806°, 38.7820°, and 48.8044°, which can be perfectly related to (–1 1 1), (1 1 1), and (–2 0 2) crystal planes, respectively. Lattice constants of the CuO phase were calculated to be a = 4.68, b = 3.42, and c = 5.12 nm. Also, diffraction peaks of Co_3_O_4_ (JCPDS Card No. 74‐2120) and SnO_2_ phases (JCPDS Card No. 88‐0287) were observed in this pattern. As shown in Figure 1(b), no amorphous phase, composed of rGO, was detected [61]. No characteristic XRD peaks arising from precursor were observed, indicating the formation of pure nanocomposite 3. The average particle size of the synthesized nanocomposite 3 was determined using the Debye–Scherrer formula to be 33.92 nm.

**FIGURE 2:**
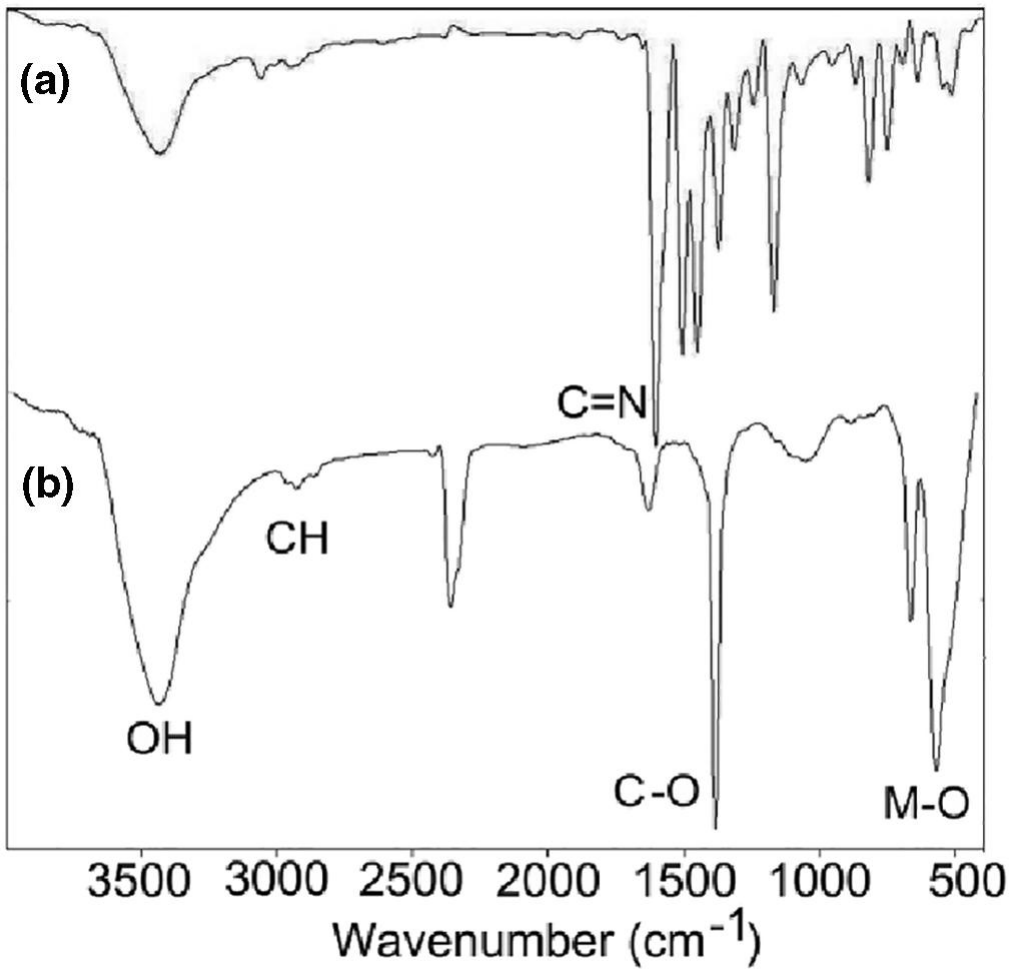
FT–IR spectra of nanocomposite 3 (b) and its corresponding precursor (a)

The FT–IR spectra of nanocomposite 3 and its corresponding precursor are shown in Figure 2. In IR spectrum of the precursor (Figure 2(a)), the band at 1607.72 cm^–1^ is related to the azomethine group (C=N) which has shifted to a lower frequency compared to the copper (II) complex of Schiff base ligand (H_2_L) (1630 cm^–1^) [57] due to the presence of cobalt and tin in the compound. In the IR spectrum of nanocomposite 3 (Figure 2(b)), a strong band was observed at around 571 cm^–1^ which can be assigned to the M–O bond (M = Cu, Co, and Sn) [57, 62]. The C–H and C–O stretching vibrations due to graphene sheets appeared at 2925 and 1383 cm^–1^, respectively [63]. Also, the appearance of bands at 3432 and 1631 cm^–1^ was assigned to the stretching and bending vibrations of the water molecules absorbed by the sample or KBr [57]. The disappearances all characteristic bands of the precursor in the IR spectrum of nanocomposite 3 demonstrated complete decomposition of the precursor into nanocomposite 3 by the solid‐state microwave‐assisted method.

**FIGURE 3:**
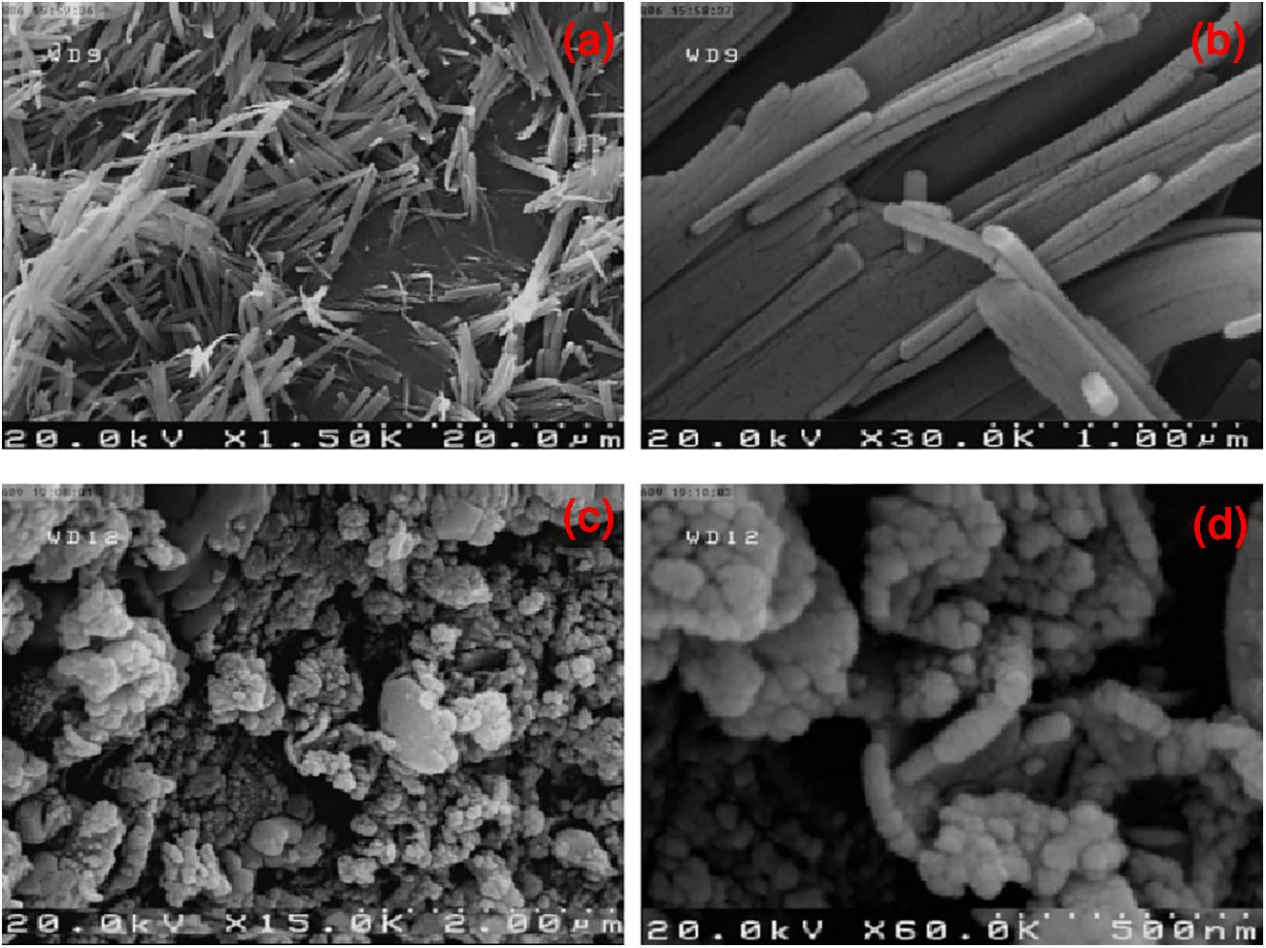
FESEM images of the corresponding precursor for nanocomposite 3 (a, b) and nanocomposite 3 (c, d) at different magnifications

The morphology of nanocomposites and their corresponding precursors were surveyed by the FESEM analysis (Figure 3). The FESEM images of the precursor of nanocomposite 3 show nanorods which were loosely aggregated (Figure 3(a) and (b)). As shown in Figure 3(c) and (d), the morphology of nanocomposite 3 is semispherical shape. The particle size distribution of the precursor and nanocomposite 3 was found to be 50–60 ± 0.8 and 30–40 ± 0.5 nm, respectively. These results are in good agreement with the XRD analysis. The striking difference between the size and shape of nanocomposite 3 with its corresponding precursor showed that their structures are quite different and confirmed that decomposition of precursor into the product particles is complete. Therefore, solid‐state microwave irradiation is a beneficial method for the synthesis of varied nano‐sized compounds in high purity. The difference between this method and the conventional heating methods results from the difference in the mechanism of the reactions. The solid‐state microwave irradiation involves converting electromagnetic energy into heat by fast kinetics of the molecules. The electromagnetic energy arises from interaction of microwave wavelengths with the reactants at the molecular level [64]. These extreme conditions lead to solid‐state decomposition reactions and the formation of nano‐sized particles in different morphologies.

**FIGURE 4:**
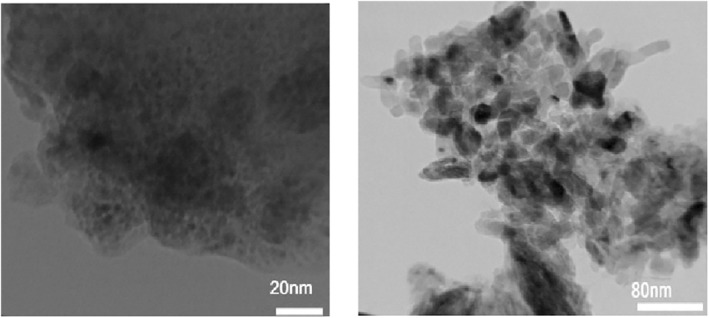
TEM images of nanocomposite 3 at different magnifications

The morphology and particle size of the synthesized nanocomposites were further investigated by TEM analysis. TEM images of nanocomposite 3 are given in Figure 4 at different magnifications. The images showed the spherical shape of particles with the particle size distribution of 30–40 ± 0.5 nm, similar to that obtained using the XRD and FESEM analysis.

**FIGURE 5:**
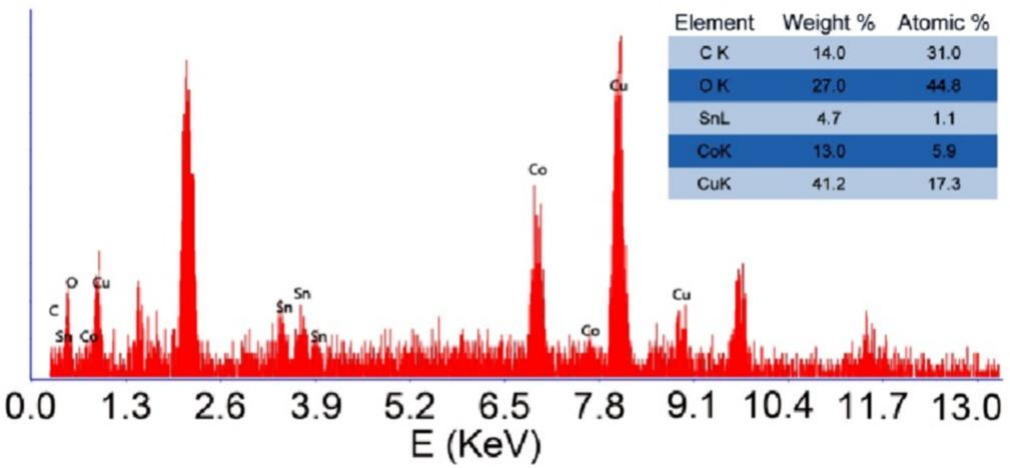
EDX spectrum of nanocomposite 3

Figure 5 shows the EDX analysis of nanocomposite 3 that confirms the presence of Co, Cu, Sn, C, and O elements in this nanocomposite. The Si and Au signals were observed due to the coating material of the instrument. Pleasingly, EDX results (inset in Figure 5) are in close agreement with the ratios used in the synthetic processes. Hence, these results show that nanocomposite 3 was prepared in high purity by the presented solid‐state microwave method.

**FIGURE 6:**
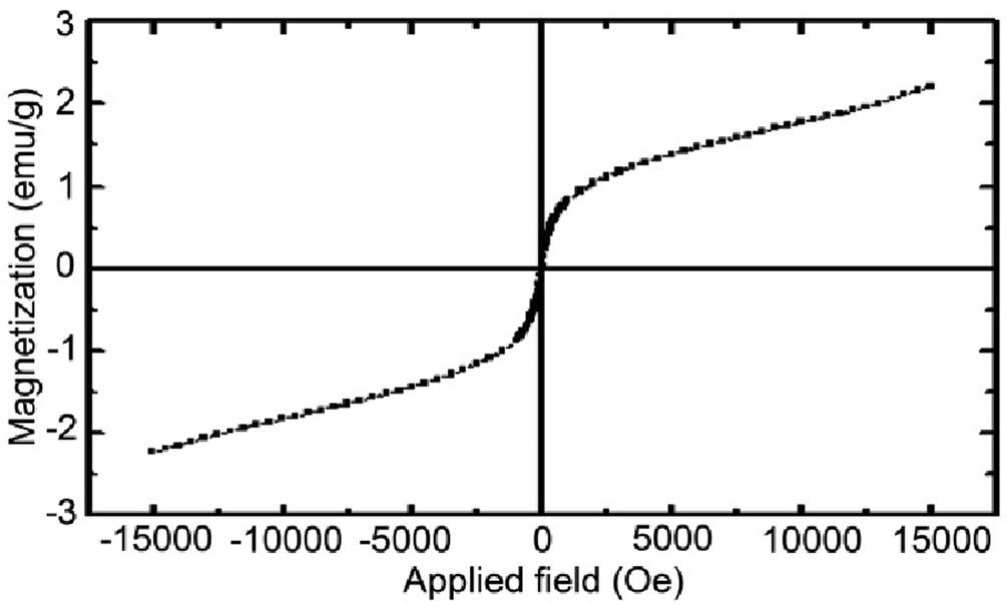
Magnetization versus applied magnetic field at room temperature for nanocomposite 3

It is believed that the magnetic property of nanomaterials is highly dependent on the type of sample shape, size of particles, magnetization direction, and synthetic method. The variation in magnetization (M) as a function of applied field (H) at room temperature for nanocomposite 3 is depicted in Figure 6 and shows a superparamagnetic behavior of the nanocomposite (Mr = 0.24 emu/g).

**FIGURE 7:**
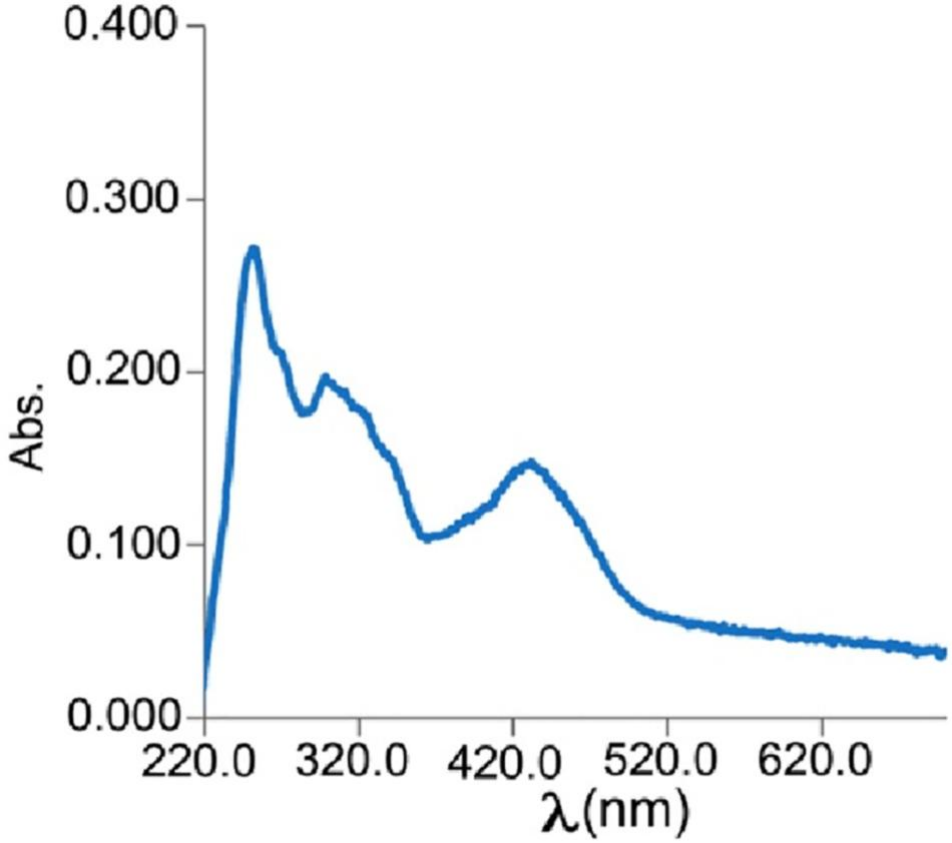
Electronic spectrum of the corresponding precursor of nanocomposite 3

The electronic property of the synthesized nanocomposites was surveyed by UV–Vis analysis. Figure 7 shows the electronic spectrum of the corresponding precursor for nanocomposite 3. The band at 247 nm was related to the π–π* transition of the aromatic rings contained in ligand structure. The bands at 393–422 nm were attributed to the MLCT (metal ligand charge transfer) and LMCT (ligand metal charge transfer) transitions [65, 66]. These bands showed a shift toward the copper (II) complex of Schiff base ligand (H_2_L) [57] due to the presence of tin and cobalt ions.

**FIGURE 8:**
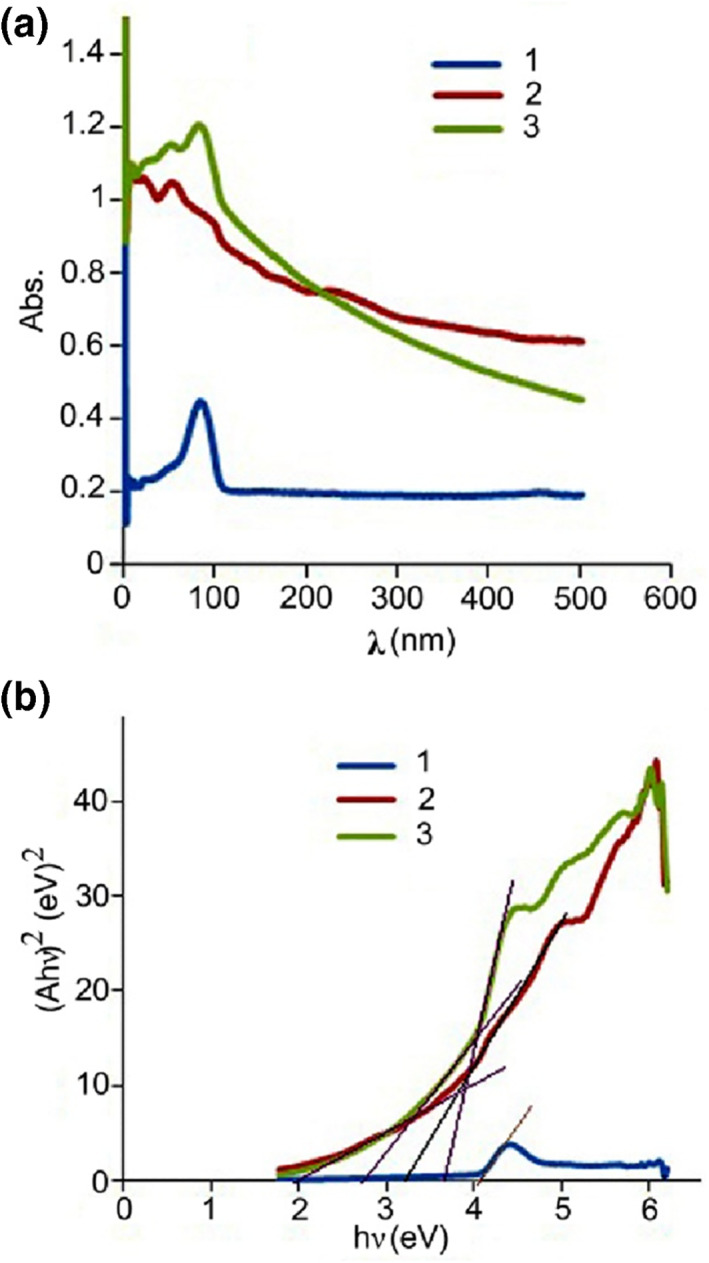
Electronic spectra of the nanocomposites 1–3 (assigned 1 (blue line), 2 (red line), and 3 (green line)) (a) and their corresponding (Ahʋ)^2^–hʋ curves (b)

Figure 8 shows electronic spectra and the (Ahυ)^2^–hυ curves of the nanocomposites 1–3. In Figure 8(a), the observed bands (<200 nm) of the nanocomposites are due to the electron transitions from Cu (3d) and O (2p) orbitals [67] and have highest intensity for nanocomposite 3. These bands showed a blue shift compared with pure CuO nanoparticles [68, 69], due to the presence of Co and Sn ions. The band gap (*E*_g_) of the synthesized nanocomposites can be obtained by the equation: (Ahυ)^2^ = B (hυ–*E*_g_), where A, hυ, and B represent the absorption coefficient, the energy of photon, and a constant depending on the type of materials, respectively. Figure 8(b) shows the (Ahυ)^2^–hυ curves for the nanocomposites 1–3. The band gaps of the nanocomposites 1–3 were estimated as 4.1, 3.7, and 3.8 eV, respectively, by extrapolation of the corresponding curves. The value of band gaps for three nanocomposites has a slight shift relative to each other, most probably due to their difference in particle size and the tin content. Based on these results, it can be predicted that the synthesized nanocomposites can be used as semiconductors.

### 3.2 Catalytic application of the nanocomposites

**SCHEME 3:**
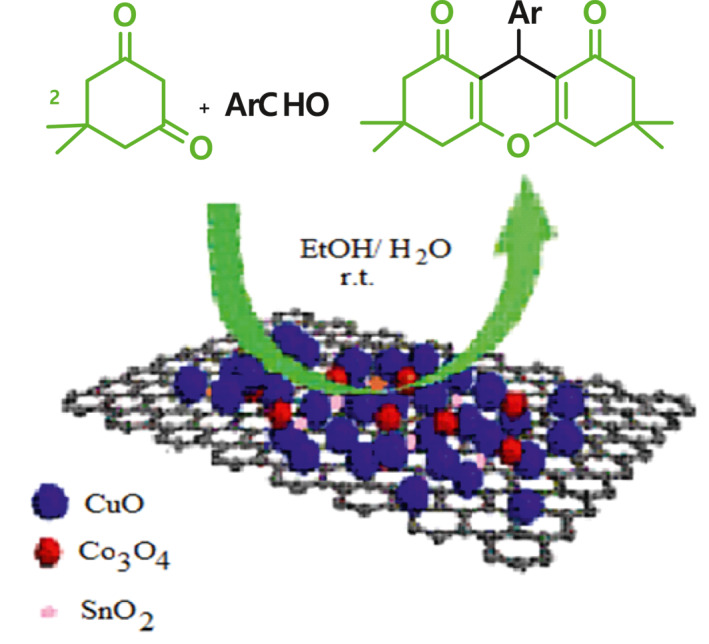
The catalytic performance of Co–Sn–Cu oxides/graphene nanocomposites in the green synthesis of 1,8‐dioxo‐octahydroxanthenes

**FIGURE 9:**
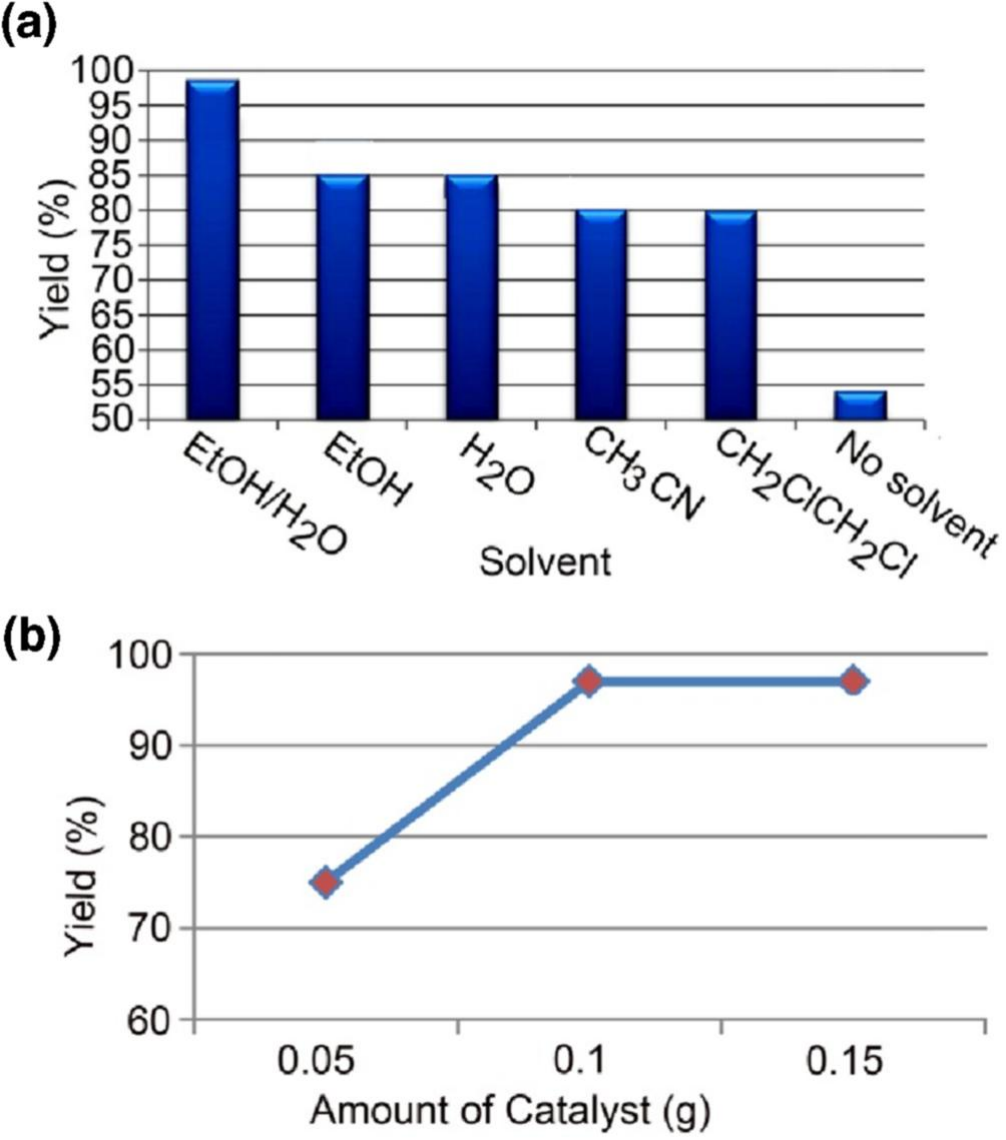
The screening of the solvent nature (a) and catalyst amount (b) on the reaction of 4‐bromobenzaldehyde (1 equivalent) with dimedone (2 equivalents) after 20 min at room temperature catalyzed by nanocomposite 3

**FIGURE 10:**
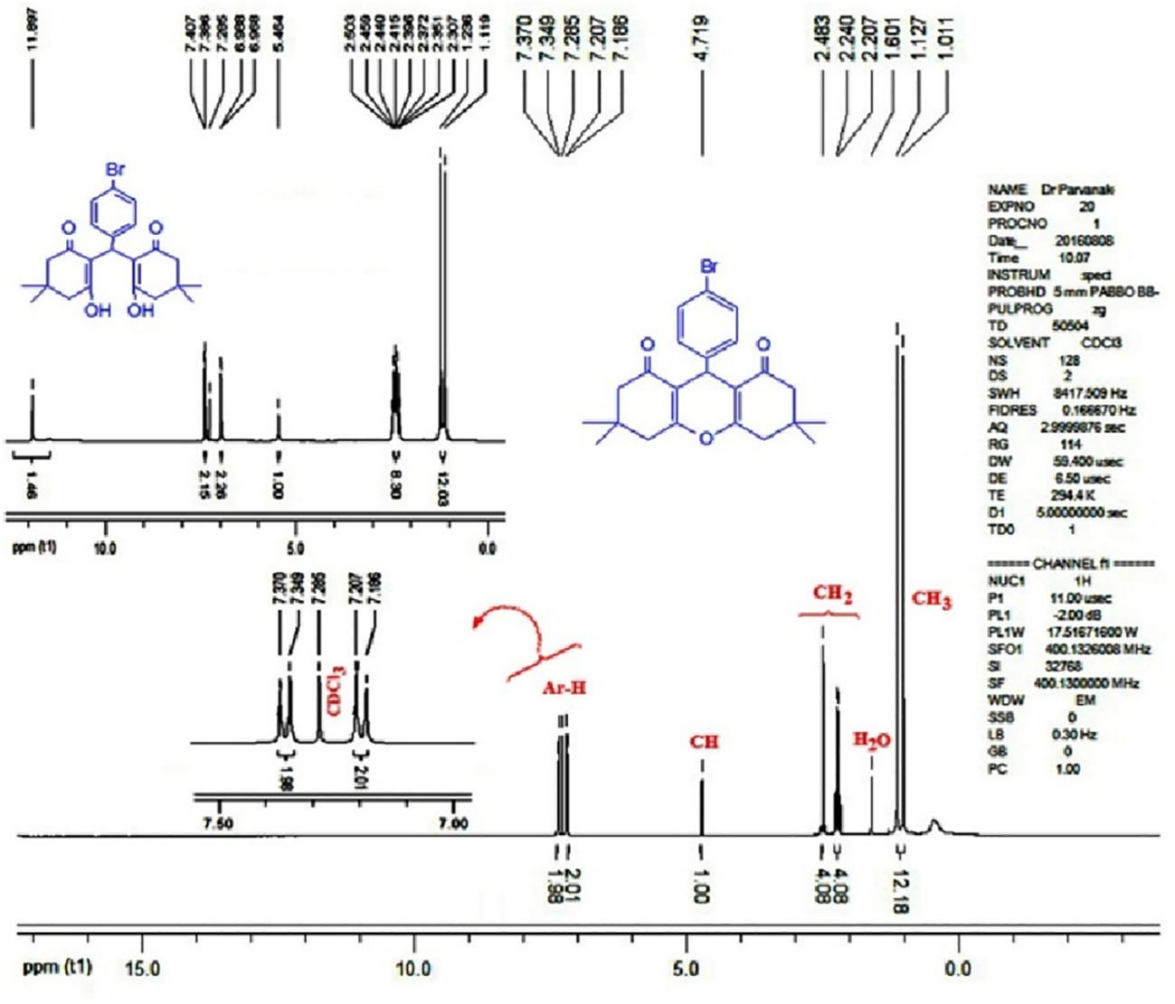
^1^H NMR spectrum of 3,4,6,7‐tetrahydro‐3,3,6,6‐tetramethyl‐9‐(4‐bromophenyl)‐2H‐xanthene‐1,8(5H,9H)‐dione

After synthesis and characterization of the nanocomposites, their catalytic activity for preparing 1,8‐dioxo‐octahydroxanthenes was examined (Scheme 3). For this purpose, the condensation reaction of 4‐bromobenzaldehyde with dimedone was chosen as a model reaction for the optimization of the reaction conditions. As it can be seen from Figure 9a,b, 3,4,6,7‐tetrahydro‐3,3,6,6‐tetramethyl‐9‐(4‐bromophenyl)‐2H‐xanthene‐1,8(5H,9H)‐dione product was obtained in 97% yield in EtOH/H_2_O (1:1) as green solvents after 20 min at room temperature in the presence of 0.1 g of nanocomposite 3. No significant difference was found between catalytic activities of nanocomposites 1–3. Thus, we typically applied this optimized condition for the reaction of a variety of aromatic aldehydes with dimedone in the presence of nanocomposite 3. The results showed that both aldehydes bearing electron‐donating, electron‐withdrawing, and halogen substituents gave the desired 1,8‐dioxo‐octahydroxanthenes in high‐to‐excellent yields (Table 1, entries 1–7, 10–12). Acid‐sensitive aldehydes such as furan‐2‐carboxaldehyde and thiophene‐2‐carboxaldehyde (entries 8, 9) reacted well and gave the corresponding products in high yields. In the absence of the catalyst, no products were formed. All the reactions were clean as indicated by TLC and the only work‐up being the easy removal of the catalyst using an external magnet and then evaporation of reaction solvent. Figure 10 shows ^1^H‐NMR spectrum of 3,4,6,7‐tetrahydro‐3,3,6,6‐tetramethyl‐9‐(4‐bromophenyl)‐2H‐xanthene‐1,8(5H,9H)‐dione.

**TABLE 1:** Preparation of 1,8‐dioxo‐octahydroxanthenes from the reaction aldehydes with dimedone using nanocomposite 3^a^

No.	Ar	Time (min)	Yield (%)^b^	mp (^o^C) (lit.) [Ref.]
1	4‐NO_2_C_6_H_5_	14	97	224–227 (226–228) [12]
2	3‐NO_2_C_6_H_5_	15	97	167–170 (170–171) [11]
3	2,4‐Cl_2_C_6_H_4_	18	96	250–253 (247–248) [11]
4	4‐CNC_6_H_5_	16	96	213–217 (217–221) [19]
5	4‐ClC_6_H_5_	19	95	225–229 (237–238) [11]
6	4‐BrC_6_H_5_	20	97	235–237 (240–241) [11]
7	C_6_H_5_	20	96	199–203 (205–206) [11]
8	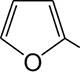	20	94	60–63 (62–63) [13]
9	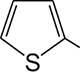	20	94	160–163 (160–165) [19]
10	4‐MeC_6_H_5_	22	93	210–213 (212–214) [10]
11	4‐HOC_6_H_5_	22	93	238–241 (244–246) [10]
12	4‐MeOC_6_H_5_	24	93	239–243 (242–245) [10]

^a^ All reactions were carried out in EtOH/H_2_O (1:1) at room temperature in the presence of nanocomposite 3 (0.1 g).

^b^ Isolated yield. All products are known compounds and were identified by comparison of their physical and spectral data with those of the authentic samples.

**SCHEME 4:**
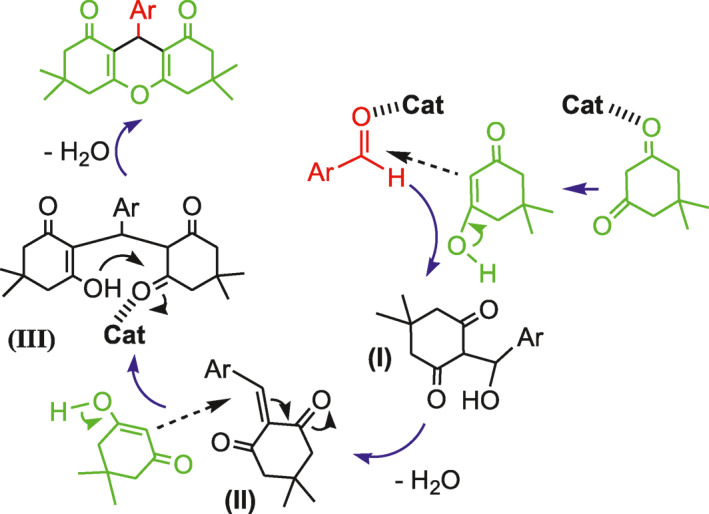
The proposed mechanism for the synthesis of 1,8‐dioxo‐octahydroxanthenes catalyzed by nanocomposite 3

A plausible mechanism for the condensation reaction of aromatic aldehydes with dimedone is as follows (Scheme 4): (1) the reaction of a molecule of enolized dimedone with aromatic aldehyde activated by the catalyst, which yields intermediate (I); (2) intermediate (I) undergoes dehydration to give the intermediate (II); (3) nucleophilic addition of second molecule of dimedone on activated intermediate (II), in the Michael addition fashion, affords uncyclized adduct 2,2’‐aryl‐methylene bis(3‐hydroxy‐2‐cyclohexene‐1‐one) (III), which produces 1,8‐dioxo‐octahydroxanthenes after intramolecular cyclodehydration [10, 11]. As it can be seen from Table 2, aromatic aldehydes bearing the electron withdrawing groups (entries 1,2) react with dimedone faster than those containing electron releasing groups (entries 10–12). According to the above‐mentioned mechanism, this could be due to the greater tendency of the intermediate II to react with dimedone in the presence of the electron withdrawing groups substituted on arylaldehydes.

**TABLE 2:** Comparison of the efficiencies of a number of different reported catalysts with that of nanocomposite 3 in the reaction of benzaldehyde with dimedone

No.	Reaction conditions	Time (min)	Yield (%)^a^
1	NHSO_4_–SiO_2_, CH_3_CN, reflux	210	95 [9]
2	SmCl_3_, solvent‐free, 120^o^C	540	98 [10]
3	[Et_3_NH][HSO_4_], solvent‐free, 100^o^C	20	94 [11]
4	Fe_3_O_4_ nanoparticles, solvent‐free, 100^o^C	30	89 [12]
5	Mg–Al hydrotalcite, H_2_O, reflux	180	85 [13]
6	Sulfated zirconia, EtOH, 70^o^C	480	95 [14]
7	Trichloromelamine, solvent‐free, 110^o^C	30	82 [15]
8	Cu(II)‐Fur‐APTES/GO^b^, EtOH/H_2_O, 50^o^C	30	95 [16]
9	Cerric ammonium nitrate, 2‐propanol, US^c^, 50^o^C	35	98 [17]
10	Ionic liquid [H‐NMP]^–^[HSO_4_]^+^ ^d^, US^c^, H_2_O, room temperature	50	86 [18]
11	Cellulose/Al_2_O_3_‐[MeIm]Cl‐XAlCl_3_ ^e^, EtOH, room temperature	25	91 [19]
12	Carbon nanotube‐BuSO_3_H, EtOH, room temperature	30	95 [20]
13	Co–Sn–Cu oxides/graphene nanocomposite 3, EtOH/H_2_O, room temperature	20	96

^a^ Isolated yield.

^b^ Furfural‐imine‐functionalized graphene oxide‐copper oxide.

^c^ Ultrasound irradiation.

^d^ 2‐Pyrodidonium hydrogen sulfate.

^e^ Cellulose aluminum oxide composite supported imidazolium chloroaluminate ionic liquid.

**FIGURE 11:**
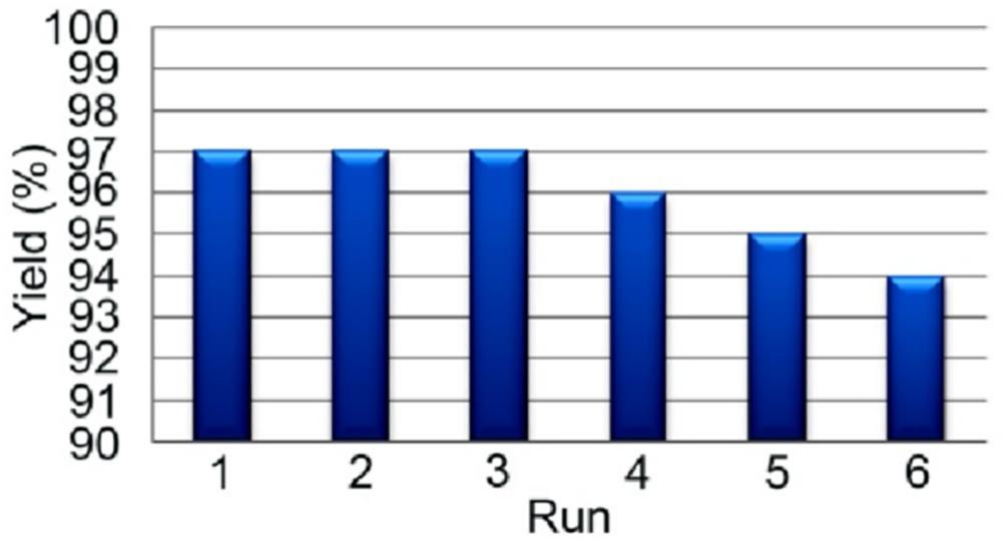
Reusability of nanocomposite 3 (0.1 g) for the reaction of 4‐bromobenzaldehyde with two equivalents of dimedone in EtOH/H_2_O (1:1) at room temperature after 20 min

To evaluate the level of reusability and recycling of the catalyst, a series of catalytic cycles were run. In each cycle, the catalyst was separated by an external magnet, washed with ethanol, and reused. As shown in Figure 11, the catalyst can be reusable with negligible loss in its activity. The activity loss is probably due to loss of the active sites of catalyst by water or reaction products during separation and washing cycles.

To find out whether the reaction takes place in the solid matrix of Co–Sn–Cu oxides/graphene nanocomposites or whether any other species (simply released in the reaction medium) are responsible in the synthesis of 1,8‐dioxo‐octahydroxanthenes, Co–Sn–Cu oxides/graphene nanocomposite (0.1 g) was added to a solution of aldehyde (1 mmol) and dimedone (2 mmol) in EtOH/H_2_O (1:1) and the mixture was stirred at room temperature for 4 h. Then, the catalyst was removed by an external magnet, and the filtrate showed no catalytic activity for the preparation of 1,8‐dioxo‐octahydroxanthenes. These observations indicate that Co–Sn–Cu oxides/graphene nanocomposites are stable under the reaction conditions; there is no danger of leaching during reactions.

Finally, the catalytic performance of nanocomposite 3 was compared with other catalysts employed for the synthesis of 1,8‐dioxo‐octahydroxanthenes (Table 2). In addition to having the general advantages attributed to heterogeneous catalysts, nanocomposite 3 has shown superiority over the other catalysts in terms of reaction temperature and time.

### 3.3 Anti‐cancer study

#### 3.3.1 Effect of nanocomposites on MCF‐7 cell proliferation

**FIGURE 12:**
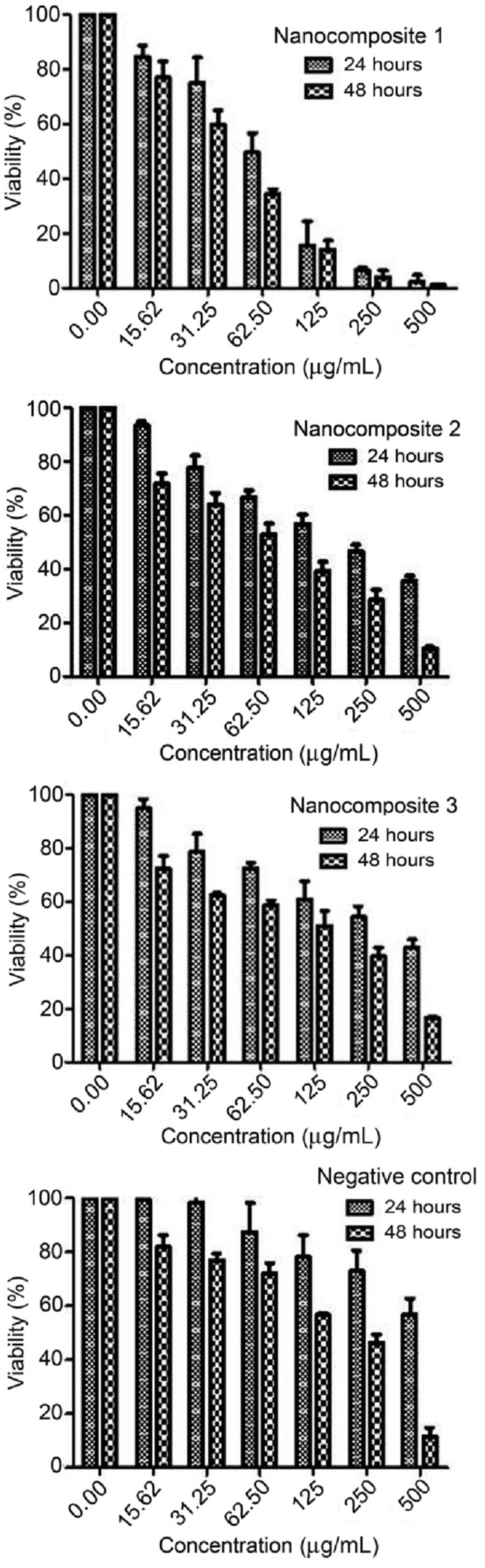
Cytotoxic effect of the nanocomposites and CuO nanoparticle as negative control on MCF‐7 cell line by the MTT assay. The data are representative of three independent experiments (mean ± SD)

**FIGURE 13:**
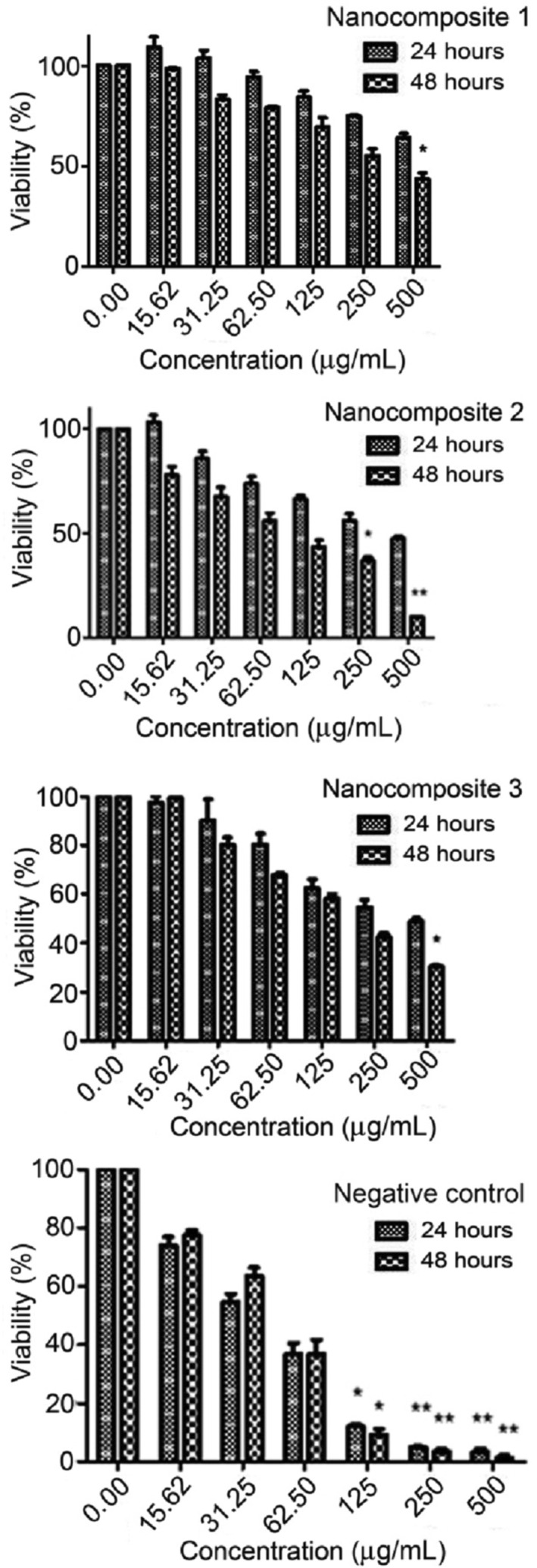
Cytotoxic effect of the nanocomposites and CuO nanoparticle as negative control on HDF cell line by the MTT assay. The data are representative of three independent experiments (mean ± SD)

Some parameters such as shape, size, dimensions, surface structure, density, chemical composition, and solubility play important role for defining the toxicity of nanocompounds [70, 71]. The effective anticancer agents are the compounds that have the most toxicity against cancerous cells whereas the lowest toxicity to normal cells. In this study, to determine the effect of nanocomposites on viability of MCF‐7 cells, cells were incubated for 24 and 48 h in the absence or presence of prepared nanocomposites at different concentrations (0–500 μg/mL). The optical density obtained from the absorption at 570 nm was converted to a percentage. Figure 12 shows the reduction of cell viability as concentration increased (dose‐dependent). A significant reduction of MCF‐7 cell viability was also observed with a time‐dependent manner at a specific concentration. Data showed that nanocomposite 1 is more powerful cytotoxic effect than others both in 24 and 48 h. IC50 (inhibition concentration) values were measured by probit analysis and were 61.82 ± 3.21, 205.73 ± 4.25, and 344.03 ± 6.32 μg/mL in 24 h, and also 43.28 ± 4.28, 78.83 ± 5.01, and 135.58 ± 9.84 μg/mL in 48 h for nanocomposites 1–3, respectively. Also, IC50 value for normal cell line was investigated. The human dermal fibroblasts (HDF) line was chosen, and values of IC50 were measured as 423.40 ± 11.53 and 444.17 ± 13.34 μg/mL for nanocomposites 2 and 3, respectively, in 24 h. In the case of nanocomposite 1, IC50 value was not observed after this time. IC50 value was investigated as 359.2 ± 13.5, 92.06 ±1.75, and 189.51±13.59 μg/mL for nanocomposites 1–3, respectively, in 48 h. The results were shown that nanocomposite 1 has less toxicity against normal cell (as shown in Figure 13. In addition, anti‐cancer property of CuO nanoparticle as negative control [57] was studied against MCF‐7 cell line. IC50 value was 206.36 ±10.21 in 48 h, whereas it was not observed in 24 h. Thus, the presence of Co and Sn in chemical structure (appropriate amount) of these nanocomposites is an effective parameter.

#### 3.3.2 Effect of the nanocomposites on MCF‐7 cell apoptosis

**FIGURE 14:**
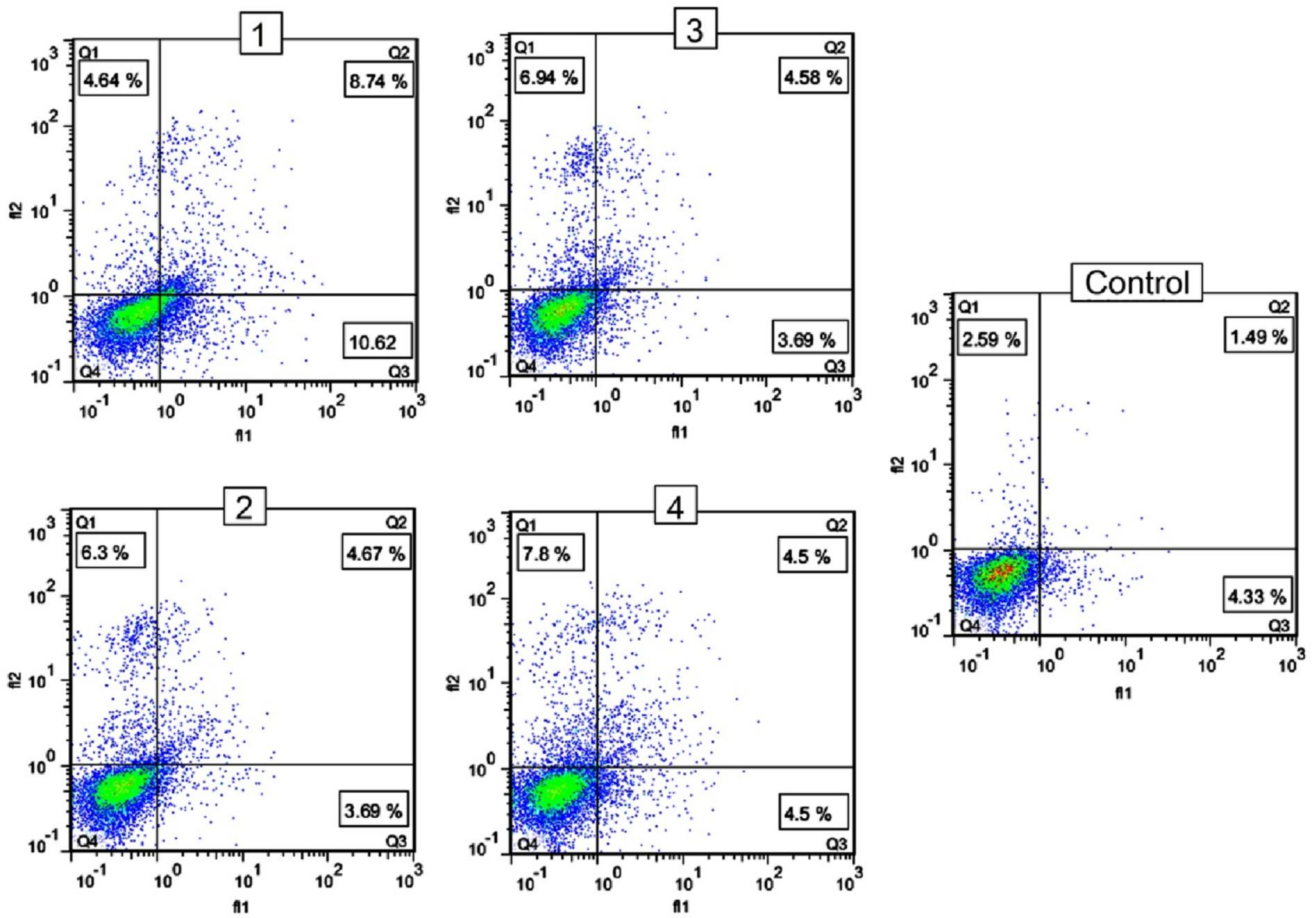
Apoptosis induction of the nanocomposites (assigned 1, 2, and 3) and CuO nanoparticle as negative control (assigned 4)

To assess whether nanocomposites could induce apoptosis in MCF‐7 cells, apoptosis induction was determined by an Annexin‐V‐FITC/PI apoptosis detection kit. MCF‐7 cells were incubated for 48 h at IC50 value. Cells that were positive just for Annexin‐V and those that were positive both for Annexin‐V and PI were considered as early and late apoptotic cells, respectively. As shown in Figure 14, nanocomposites could induce apoptosis in treated cell and nanocomposite 1 was more powerful in inducing apoptosis, compared with other nanocomposites and CuO nanoparticle.

**FIGURE 15:**
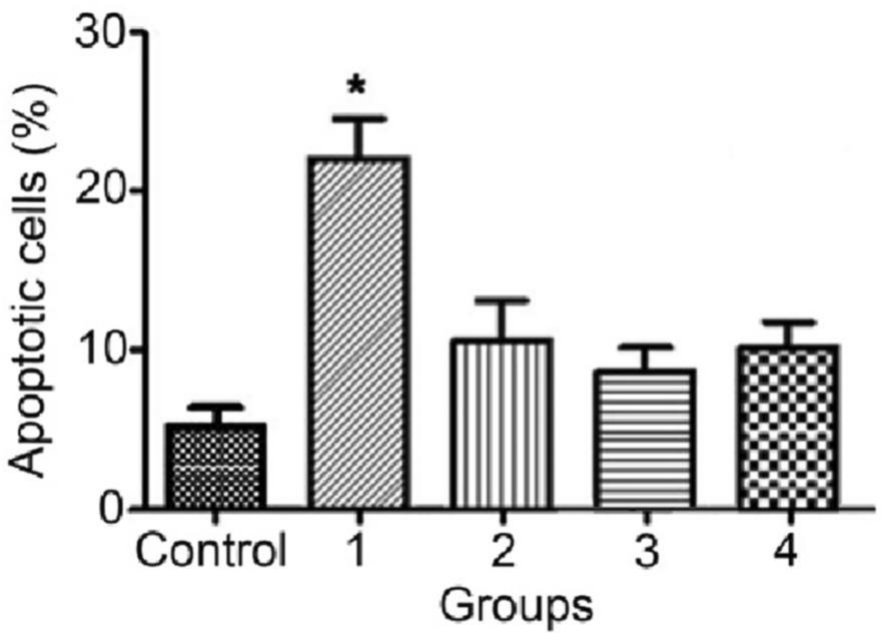
Effect of the nanocomposites (assigned 1, 2, and 3) and CuO nanoparticle as negative control (assigned 4) on apoptosis after 48 h. The percentage of cells in early and late apoptosis was considered as apoptosis

Finally, cells were exposed to IC50 value of nanocomposites for 48 h. Dot plots show the results of a representative apoptosis assay. The number of cells in apoptosis indicated as percentage relative to the total cell number. Treated cells with nanocomposites at IC50 value showed a significant increase in the amount of apoptotic cells compared with the control group just in nanocomposite 1 (*P<0.05) (Figure 15).

So far, many investigations were done based on metallic nanoparticles (metal oxides, metallic nanocomposites, magnetic nanoparticles, graphene‐based nanoparticles etc.) to find out effective anticancer drugs that play an important role in the apoptosis [72, 73, 74, 75, 76, 77]. The presence of anionic phospholipids in cancer cells causes negative zeta potential that absorb into positively charged nanoparticles by strong electrostatic interaction [78, 79]. The nanoparticles cause the inflammation, damage of DNA or cell membrane, and eventually cell death occurs [80]. In this work, we prepared Co–Sn–Cu oxides/graphene nanocomposites, for the first time, via solid‐state microwave method as a new, simple, and fast strategy and their anti‐cancer effects in MCF‐7 cells were investigated via apoptosis induction. The results showed that nanocomposite 1 was more potent than copper nanoparticles (CuNP, IC50 = 85 µg/mL) [81], silver nanoparticles (IC50 = 90 µg/mL) [82], Fe_3_O_4_/Ag nanocomposite (IC50 = 135 μg/mL) [83], and copper sulfide quantum dots (CuSQDs) loaded nano‐GO (IC50 = 100 µg/mL) [84]. The superiority of our introduced nanocomposites may be due to different factors such as type and ratio of elements that resided in nanocomposite, type of precursor, as well as synthetic method. We anticipate that the solid‐state microwave method, used for the preparation of the present nanocomposites 1–3, would be the newly developed route for the synthesis of different anticancer drugs based on nanostructured materials. In addition, as mentioned in the Introduction section, graphene and its derivatives have supreme physicochemical and pharmaceutical properties, which make them good candidate for research in cancer therapy, recently [85, 86]. The biodegradation of graphene nanomaterials by microbes and enzymes has also been documented [87, 88]. Therefore, considering the presence of the Co–Sn–Cu oxides on graphene surface in the presented Co–Sn–Cu oxides/graphene nanocomposites and obtaining very good results in the context of anticancer activity at this stage, we predict that these nanocomposites are potential and promising materials for the preparation of anticancer drugs after taking part in clinical trials.

## 4 CONCLUSIONS

In this work, we proposed solid‐state microwave irradiation as a simple, efficient, safe, fast, and environmental friendly method for the synthesis of pure Co–Sn–Cu oxides/graphene nanocomposites. This method did not require fuel, solvent, surfactant, any expensive materials, and any complex instruments. The nanocomposites were used as green catalysts for preparing 1,8‐dioxo‐octahydroxanthenes at room temperature. The catalysts can be easily separated from reaction media using an external magnet and are reusable. Simple work‐up procedure, easy handling of the catalyst, high yields, and short reaction times are the other obvious advantages of the present method. Following these results, the researchers further investigated anti‐cancer activity of the nanocomposites. The results of our study showed that nanocomposites can silence cell proliferation with a time‐ and dose‐dependent manner. Also, the results demonstrated that nanocomposites can induce apoptosis in breast cancer cell line which is an advantageous effect of anti‐cancer therapy. The results indicated that nanocomposite 1 was a more powerful apoptosis inducer than other nanocomposites in MCF‐7 cells. On the basis of the results, following 48 h incubation, it derived almost 30% of cells to apoptosis at IC50 value concentrations. Pleasingly, photocatalyst properties of the synthesized nanocomposites are under investigation and will be reported in due course.
